# Development and validation of a nomogram for differentiating bacterial from non-bacterial necrotizing pneumonia in children: a retrospective cohort study

**DOI:** 10.3389/fmed.2026.1854223

**Published:** 2026-09-01

**Authors:** Mengyao Zhang, Jiafeng Zheng, Hanquan Dong, Bo Wang, Yongsheng Xu

**Affiliations:** Department of Pediatric Respiratory Medicine, Children's Hospital, Tianjin University/Tianjin Children's Hospital, Tianjin, China

**Keywords:** bacterial, children, etiological differentiation, necrotizing pneumonia, nomogram, non-bacterial, prediction model

## Abstract

**Background:**

Necrotizing pneumonia (NP) in children requires etiology-dependent treatment, yet early etiological differentiation remains challenging due to the low sensitivity and slow turnaround of conventional microbiological methods. This study aimed to develop and validate a nomogram for predicting non-bacterial NP using routinely available clinical parameters.

**Methods:**

This single-center retrospective study enrolled children (<18 years) with contrast-enhanced computed tomography (CT)–confirmed NP at Tianjin Children’s Hospital (January 2020–January 2025). Patients were classified as bacterial NP (BNP) or non-bacterial NP (NBNP) based on pathogen identification via culture, polymerase chain reaction (PCR), or targeted next-generation sequencing, and randomly split into training and validation sets (7:3). Predictors were selected by univariable screening followed by stepwise multivariable logistic regression, and a nomogram was constructed. Performance was assessed using receiver operating characteristic (ROC) analysis, calibration plots, and decision curve analysis.

**Results:**

Of 149 children (76 BNP, 73 NBNP; training *n* = 104, validation *n* = 45), three independent predictors of NBNP were identified: fever duration (odds ratio [OR] = 4.580, *p* = 0.004), CRP (OR = 0.272, *p* = 0.009), and white blood cell count (WBC) (OR = 0.257, *p* = 0.025). The nomogram achieved an AUC of 0.816 (95% CI: 0.735–0.896) in the training cohort. Bootstrap internal validation (1,000 resamples) of the validation cohort yielded a bias-corrected AUC of 0.846 (95% CI, 0.732–0.959), confirming stable discriminative performance. Calibration was satisfactory (Hosmer–Lemeshow *p* = 0.857 and 0.738), and decision curve analysis demonstrated positive net benefit.

**Conclusion:**

A parsimonious nomogram based on fever duration, WBC count, and CRP level offers a practical bedside tool for early etiological differentiation of NP in children, potentially guiding timely pathogen-directed therapy before microbiological confirmation. External multicenter prospective validation is warranted.

## 1 Introduction

Necrotizing pneumonia (NP) in children is a severe complication of community-acquired pneumonia (CAP), with approximately 3% of CAP cases developing pulmonary necrosis (1). Treatment is fundamentally pathogen-dependent: bacterial NP (BNP) requires intensified antimicrobial therapy (e.g., vancomycin or linezolid-based regimens), whereas non-bacterial NP (NBNP)—caused by pathogens such as *Mycoplasma pneumoniae* or viruses—relies primarily on macrolides combined with corticosteroids and intravenous immunoglobulin. Misidentification of the etiology can lead directly to treatment failure; thus, early etiological differentiation is essential for optimizing outcomes.

Although NP has been predominantly attributed to toxin-producing bacteria, reports of non-bacterial NP caused by *M. pneumoniae*, adenovirus, and influenza virus have increased in recent years (2–4), suggesting that non-bacterial etiologies may be substantially underestimated. However, early differentiation remains challenging. Conventional blood cultures yield positivity rates of only 10–12%, with turnaround times of 24–72 h or longer (4). Molecular detection methods offer superior sensitivity but are not universally available, serological assays are limited by diagnostic window periods, and radiological findings (consolidation with cavitation) are shared by both etiological types, offering limited discriminatory value.

Existing prediction models in NP have focused on identifying which children will develop NP (e.g., predicting progression from refractory *Mycoplasma pneumoniae* pneumonia to NP) (5), rather than on classifying the etiology of established NP. Consequently, clinicians still lack objective tools for rapid etiological determination once NP has been confirmed or is strongly suspected. To date, no prediction model has been developed to address this clinical gap.

This study is novel in targeting etiological classification—rather than occurrence prediction—of pediatric NP. Using retrospective cohort data, we constructed and internally validated a nomogram based on three routinely available parameters—white blood cell count (WBC), C-reactive protein (CRP), and fever duration—to provide a practical bedside tool for early differentiation between BNP and NBNP before microbiological confirmation becomes available.

## 2 Methods

### 2.1 Study design and patient selection

We performed a retrospective observational analysis at a single tertiary institution (Department of Respiratory Medicine, Tianjin Children’s Hospital). The electronic medical records of all pediatric inpatients who received a discharge diagnosis of necrotizing pneumonia (NP) during the period from January 2020 through January 2025 were systematically screened.

Eligible participants met all of the following criteria: (1) younger than 18 years at admission; (2) NP confirmed by contrast-enhanced chest computed tomography (CT) findings; (3) an established causative pathogen enabling unambiguous assignment to either the bacterial necrotizing pneumonia (BNP) or non-bacterial necrotizing pneumonia (NBNP) category. Patients were excluded if they had: (1) any form of congenital or acquired immunodeficiency, or were on immunosuppressive agents at the time of diagnosis; (2) pre-existing chronic cardiopulmonary disorders such as bronchopulmonary dysplasia, congenital cardiac anomalies, interstitial lung disease, or unexplained chronic cough; (3) missing key clinical or laboratory variables that precluded model construction; (4) concurrent detection of both bacterial and non-bacterial organisms (mixed infections).

No formal sample-size estimation was conducted prior to data collection. Instead, model adequacy was verified *post hoc* using the events per variable (EPV) rule, whereby at least 10 outcome events were required for each candidate predictor entered into the logistic regression framework.

Ethical approval was granted by the Institutional Review Board of Tianjin Children’s Hospital (approval number: W-2026-058).

### 2.2 Diagnostic criteria and definitions

The radiological diagnosis of NP was established when contrast-enhanced chest CT revealed disruption of the normal lung parenchymal enhancement pattern, manifesting as regions of non-enhancing devitalized tissue, liquefactive change, or intrapulmonary cavitation within an area of consolidation, consistent with criteria described previously (6). BNP was diagnosed when at least one bacterial organism was recovered or detected from sputum or bronchoalveolar lavage fluid (BALF) by any of the following approaches: conventional aerobic/anaerobic culture, nucleic acid–based respiratory pathogen panel testing, or targeted next-generation sequencing (tNGS).

NBNP was defined when a non-bacterial organism was the sole identified pathogen, with no concurrent evidence of bacterial co-infection. The predominant non-bacterial agents comprised *Mycoplasma pneumoniae*, *Chlamydia pneumoniae*, adenovirus, and human metapneumovirus; their identification relied on polymerase chain reaction (PCR) assays performed on sputum or BALF samples. Fever duration was operationally defined as the interval (in days) between the first documented day of fever and the date of hospital admission (or the date on which NP was initially identified on CT, whichever came first).

For the purpose of regression modeling, three continuous or semi-continuous variables were re-coded as ordinal categories. Fever duration was stratified into three tiers: short (1–5 days), intermediate (6–10 days), and prolonged (≥11 days). WBC count was categorized as low (≤4 × 10^9^/L), normal (4–12 × 10^9^/L), or elevated (>12 × 10^9^/L). CRP level was grouped as normal (≤8 mg/L), mildly elevated (8–50 mg/L), or markedly elevated (>50 mg/L).

Despite employing multiple detection modalities, some degree of etiological misclassification cannot be entirely excluded due to the suboptimal sensitivity of sputum culture. A sensitivity analysis restricted to patients with BALF culture– or tNGS-confirmed diagnoses was performed to assess model robustness (Supplementary Figure 1).

### 2.3 Data collection

For each enrolled patient, the following variables were retrieved from the hospital’s electronic record system. Demographic data included sex, age, height, and weight. Clinical variables comprised peak temperature, duration of fever and cough, presence of diminished breath sounds, auscultatory abnormalities, and pertinent medical history. A comprehensive panel of laboratory indices was recorded: WBC, neutrophil percentage (N%), CRP, hemoglobin (Hb), platelet count (PLT), procalcitonin (PCT), D-dimer, erythrocyte sedimentation rate (ESR), serum ferritin (FER), immunoglobulin E (IgE), interleukin-6 (IL-6), creatine kinase-MB (CK-MB), creatine kinase (CK), alanine aminotransferase (ALT), aspartate aminotransferase (AST), lactate dehydrogenase (LDH), lactic acid, and prealbumin. Imaging features abstracted from CT reports encompassed laterality (unilateral or bilateral disease), presence of atelectasis, and pleural effusion.

### 2.4 Cohort partition

Patients were allocated to a derivation (training) set and an internal validation set in a 7:3 ratio through stratified randomization based on the outcome (BNP versus NBNP), thereby maintaining balanced class proportions across both subsets. A predetermined random seed ensured that the partition was fully reproducible. The derivation set served for model building, whereas the held-out validation set was used exclusively for performance assessment.

### 2.5 Statistical analysis

Distributional normality of continuous variables was examined with the Shapiro–Wilk test. Normally distributed data were summarized as mean ± SD and compared via the independent-samples *t*-test; skewed data were reported as median (interquartile range) and compared using the Mann–Whitney *U* test. Categorical variables were described as counts (percentages) and analyzed with the chi-square or Fisher’s exact test where appropriate.

Candidate predictors were first screened through univariable logistic regression. Those attaining *p* < 0.05 were carried forward into a multivariable logistic regression model employing stepwise selection: a variable entered the model if the likelihood-ratio test *P* was below 0.05 and was eliminated if the *p* value rose above 0.10. The resulting final model was translated into a nomogram to offer a graphical tool for estimating the individual probability of NBNP.

Model performance was appraised across three domains in both the derivation and validation sets. For discrimination, receiver operating characteristic (ROC) analysis was conducted; the AUC with its 95% confidence interval was computed, and the threshold maximizing the Youden index was selected as the optimal cutoff. Given the modest size of the validation subset (*n* = 45), Bootstrap resampling (1,000 iterations) was additionally performed to derive a bias-corrected AUC and corresponding confidence interval, thereby providing a more robust gauge of predictive stability. Calibration was examined through graphical calibration plots supplemented by the Hosmer–Lemeshow test (*p* > 0.05 denoting acceptable fit). Clinical usefulness was quantified via decision curve analysis (DCA), which plots net benefit against a range of clinically plausible threshold probabilities. To evaluate resilience against possible etiological misclassification stemming from lower-sensitivity sputum-based testing, a sensitivity analysis was carried out by re-estimating the model in the subset of patients whose diagnoses were confirmed exclusively by BALF culture or tNGS.

All analyses were executed in R version 4.3.1 (R Foundation for Statistical Computing, Vienna, Austria). Key packages included rms (nomogram construction and calibration), pROC (ROC analysis), and rmda (decision curve analysis). Statistical significance was set at a two-tailed *p* < 0.05.

## 3 Results

### 3.1 Baseline characteristics of the study population

The final analytic cohort comprised 149 pediatric NP cases, of which 76 were assigned to the BNP group and 73 to the NBNP group. Following stratified randomization at a 7:3 ratio, 104 patients constituted the derivation set and 45 formed the internal validation set. A detailed comparison of demographic, clinical, laboratory, and imaging features between the two etiological groups is presented in Table 1.

**Table 1:** Comparison table of baseline data between BNP and NBNP groups.

Characteristics	BNP (*n* = 76)	NBNP (*n* = 73)	*p*
Gender			0.593
Female	28 (36.84%)	31 (42.47%)	
Male	48 (63.16%)	42 (57.53%)	
Age	8.00 [7.00;10.00]	9.00 [6.00;10.00]	0.789
Weight	30.00 [23.10;42.00]	39.50 [23.10;43.70]	0.332
Height	135.00 [113.00;155.00]	136.00 [112.00;152.00]	0.892
Fever peak	38.75 [37.98;39.30]	38.80 [38.70;38.90]	0.436
Fever duration	8.00 [6.75;9.25]	14.00 [10.00;16.00]	<0.001
Cough duration	18.50 [12.75;27.00]	20.00 [14.00;26.00]	0.678
Eczema history			0.209
No	39 (51.32%)	29 (39.73%)	
Yes	37 (48.68%)	44 (60.27%)	
Rhinitis history			1.000
No	37 (48.68%)	36 (49.32%)	
Yes	39 (51.32%)	37 (50.68%)	
Abnormal lung auscultation			1.000
No			
Yes	76 (100.00%)	73 (100.00%)	
Unilateral			0.824
No	33 (43.42%)	34 (46.58%)	
Yes	43 (56.58%)	39 (53.42%)	
Atelectasis			0.531
No	70 (92.11%)	64 (87.67%)	
Yes	6 (7.89%)	9 (12.33%)	
Pleural effusion			0.69
No	68 (89.47%)	62 (84.93%)	
Yes	8 (10.53%)	11 (15.07%)	
Decreased breath sounds			0.824
No	33 (43.42%)	34 (46.58%)	
Yes	43 (56.58%)	39 (53.42%)	
WBC	14.75 [13.70;16.27]	6.95 [5.50;8.52]	<0.001
N	75.10 [73.30;76.03]	64.60 [60.00;69.60]	<0.001
CRP	14.79 [5.96;25.90]	6.80 [2.50;13.60]	0.002
Hb	128.14 (11.03)	127.63 (12.99)	0.795
plt	272.50 [226.50;332.75]	298.00 [244.00;367.00]	0.096
pct	0.12 [0.11;0.13]	0.15 [0.07;0.30]	0.083
La	2.54 [2.14;2.98]	2.51 [2.19;2.83]	0.494
Pa	0.11 [0.10;0.14]	0.12 [0.10;0.14]	0.404
LDH	320.00 [284.75;380.00]	320.00 [287.00;380.00]	0.883
AST	23.00 [18.00;28.00]	23.00 [18.00;28.00]	0.611
ALT	14.00 [9.00;15.00]	14.60 [12.00;24.00]	0.468
CK	87.00 [67.00;120.50]	85.00 [45.00;125.00]	0.354
CKMB	9.00 [6.00;12.00]	8.00 [7.00;9.00]	0.071
IL-6	55.53 [36.95;61.41]	55.28 [36.95;60.35]	0.665
LgE	122.65 [52.12;344.60]	143.60 [62.27;355.30]	0.731
FER	257.25 [249.85;261.92]	321.00 [149.50;425.00]	0.150
ESR	27.00 [23.75;33.00]	30.00 [22.00;44.00]	0.250
D-D	0.50 [0.36;0.82]	0.50 [0.22;1.50]	0.721
Fg	4.32 [3.80;4.66]	3.91 [3.28;4.83]	0.199

The BNP and NBNP groups were well balanced with regard to sex, age, body weight, height, peak temperature, duration of cough, history of eczema, and history of rhinitis (all *p* > 0.05). Abnormal auscultatory findings were universally present in both groups. Similarly, the frequency of unilateral pulmonary involvement, atelectasis, pleural effusion, and diminished breath sounds did not differ significantly between the two groups (all *p* > 0.05).

In contrast, notable between-group differences emerged for several clinical and laboratory indices. Children with NBNP demonstrated markedly longer fever duration than those with BNP (median 14.00 [10.00–16.00] vs. 8.00 [6.75–9.25] days, *p* < 0.001). At the laboratory level, the NBNP group showed substantially lower WBC (6.95 [5.50–8.52] vs. 14.75 [13.70–16.27] × 10^9^/L, *p* < 0.001), reduced neutrophil percentages (64.60 [60.00–69.60] vs. 75.10 [73.30–76.03]%, *p* < 0.001), and lower CRP concentrations (6.80 [2.50–13.60] vs. 14.79 [5.96–25.90] mg/L, *p* = 0.002). The remaining laboratory markers—hemoglobin, platelet count, procalcitonin, lactic acid, prealbumin, LDH, AST, ALT, CK, CK-MB, IL-6, IgE, ferritin, ESR, D-dimer, and fibrinogen—were comparable across groups (all *p* > 0.05).

### 3.2 Identification of independent predictors for NBNP

To identify candidate variables associated with NBNP, univariable logistic regression was performed on all collected parameters. Those achieving *p* < 0.05 were forwarded to a multivariable model incorporating stepwise selection. Full results of both analytic stages appear in Table 2.

**Table 2:** Univariate and multivariate logistic regression analysis was performed on BNP and NBNP groups.

Characteristics	Univariate logistic regression		Multivariate logistic regression	
	OR95%CI	*p*	OR95%CI	*p*
Breath sounds low	1.182 (0.546–2.571)	0.672	0.590 (0.130–2.343)	0.468
Pleural effusion	0.84 (0.246–2.871)	0.776	1.018 (0.150–7.997)	0.985
Atelectasis	1.833 (0.538–7.262)	0.349	2.155 (0.273–21.82)	0.483
Cough duration	1.317 (0.522–3.445)	0.563	2.630 (0.542–15.31)	0.249
Fever duration	4.914 (2.479–10.93)	0.004	4.580 (1.724–14.44)	0.004
Thermal peak	0.667 (0.296–1.476)	0.321	0.545 (0.139–2.027)	0.367
Age	0.862 (0.52–1.417)	0.559	1.022 (0.429–2.394)	0.959
Gender	0.874 (0.396–1.912)	0.735	0.586 (0.143–2.242)	0.441
CRP	0.35 (0.191–0.609)	0.009	0.272 (0.094–0.683)	0.009
N	0.418 (0.188–0.861)	0.023	0.733 (0.212–2.463)	0.613
WBC	0.18 (0.08–0.36)	0.025	0.257 (0.068–0.781)	0.025
D-D	1.207 (0.702–2.115)	0.499	1.074 (0.406–2.843)	0.883
ESR	1.173 (0.642–2.161)	0.604	1.351 (0.533–3.559)	0.528
FER	1.235 (0.628–2.52)	0.545	2.044 (0.661–6.950)	0.225
LgE	1.106 (0.678–1.834)	0.688	1.180 (0.542–2.562)	0.667
IL-6	1.347 (0.868–2.142)	0.193	1.115 (0.539–2.397)	0.769
CKMB	1.32 (0.354–5.447)	0.682	2.751 (0.311–28.31)	0.371
CK	0.806 (0.487–1.309)	0.385	0.737 (0.282–1.838)	0.517
ALT	0.807 (0.363–1.76)	0.587	1.357 (0.336–6.314)	0.682
AST	1.155 (0.564–2.465)	0.696	0.678 (0.205–2.287)	0.521
LDH	0.784 (0.35–1.748)	0.551	0.697 (0.174–2.597)	0.595
la	1.007 (0.563–1.813)	0.982	0.998 (0.329–2.891)	0.998
pct	0.975 (0.433–2.184)	0.951	0.672 (0.158–2.775)	0.581
plt	2.241 (0.991–5.244)	0.056	2.256 (0.599–8.959)	0.232
Hb	1.647 (0.526–5.717)	0.403	1.215 (0.243–6.746)	0.815

The stepwise procedure retained three variables as independent determinants of NBNP in the final model: fever duration (OR = 4.580, 95% CI: 1.724–14.44, *p* = 0.004), CRP (OR = 0.272, 95% CI: 0.094–0.683, *p* = 0.009), and WBC (OR = 0.257, 95% CI: 0.068–0.781, *p* = 0.025). These estimates indicated that a longer febrile course increased the likelihood of a non-bacterial etiology, whereas higher CRP and WBC values pointed toward bacterial causation.

### 3.3 Construction of the predictive nomogram

**Figure 1:**
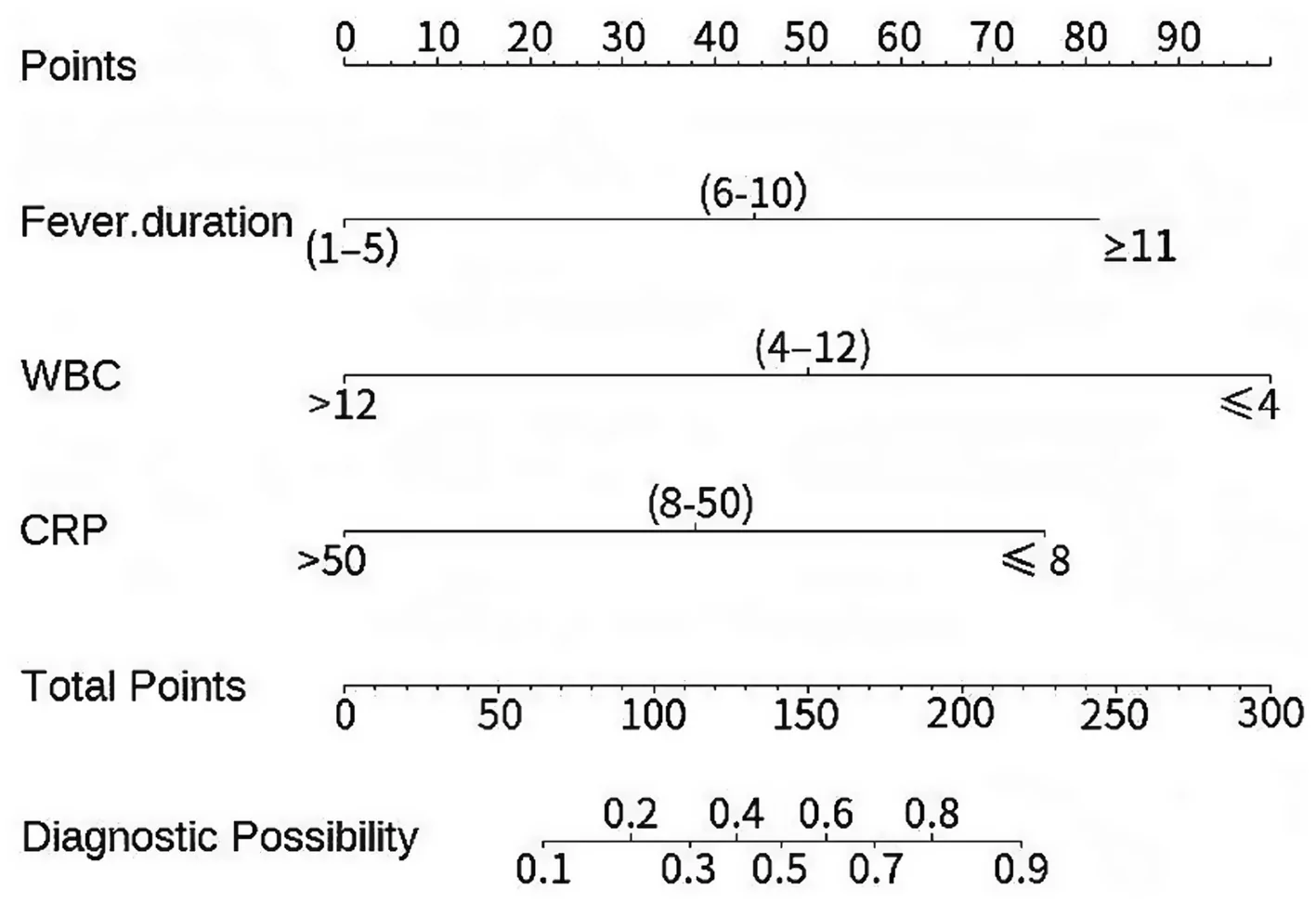
Nomogram constructed by a clinical prediction model for predicting non-bacterial necrotizing pneumonia.

Based on the three retained predictors, a graphical nomogram was generated to translate the regression coefficients into an intuitive clinical scoring tool for estimating the probability of NBNP (Figure 1). All three predictors were incorporated as three-tier ordinal categories. For fever duration, level 1 corresponded to 1–5 days, level 2 to 6–10 days, and level 3 to ≥11 days. WBC was categorized as level 1 (≤4 × 10^9^/L), level 2 (4–12 × 10^9^/L), and level 3 (>12 × 10^9^/L). CRP was classified as level 1 (≤8 mg/L), level 2 (>8–50 mg/L), and level 3 (>50 mg/L). To use the nomogram at the bedside, the clinician identifies the patient’s value for each predictor on the respective axis, draws a vertical projection to the uppermost “Points” ruler to read the corresponding partial score, sums the three partial scores to obtain total points, and finally maps the total onto the lower “Diagnostic Possibility” axis to derive the estimated probability of NBNP.

### 3.4 Validation of the nomogram

#### 3.4.1 Nomogram performance was assessed in both the derivation and validation cohorts with respect to discrimination and calibration

**Figure 2:**
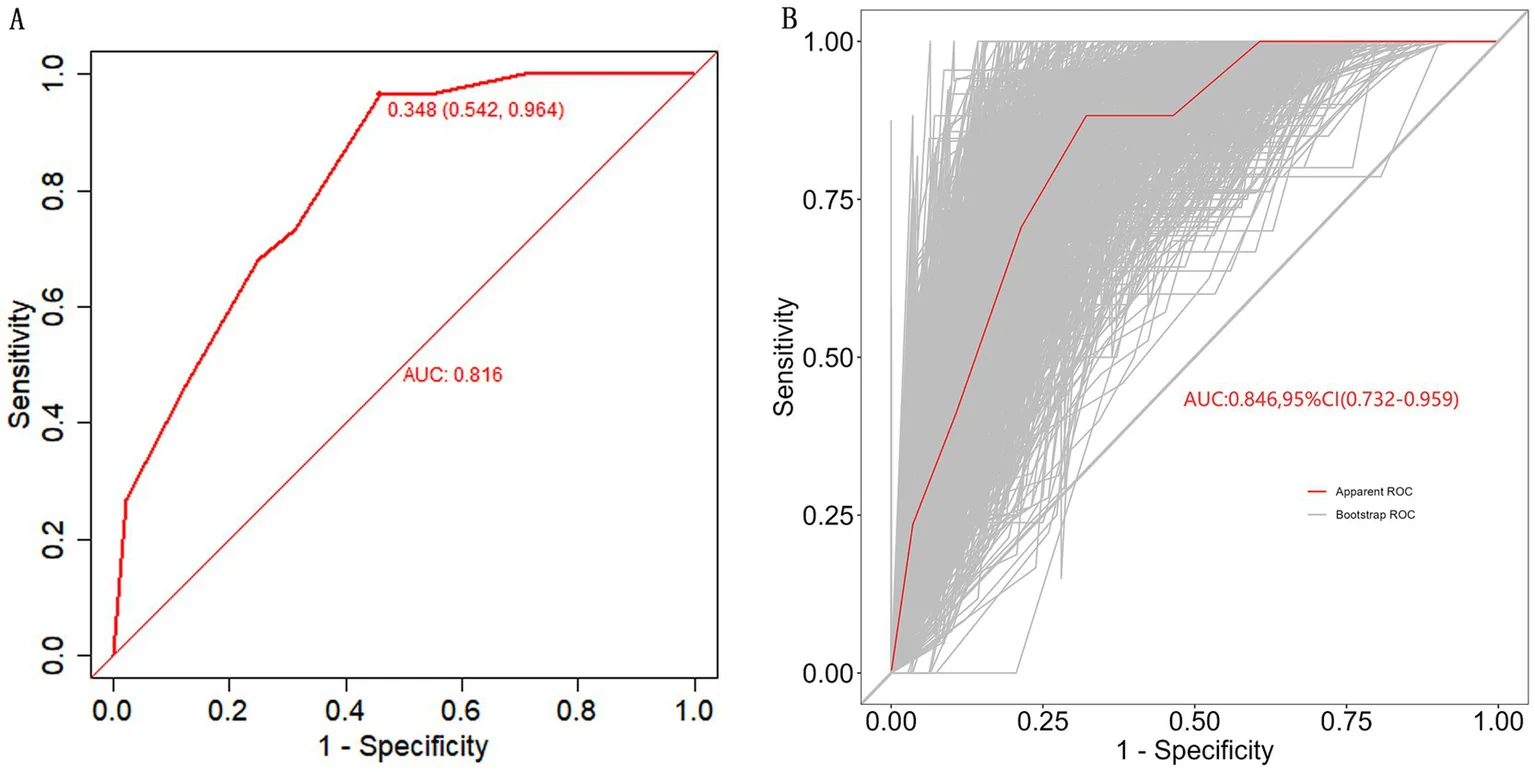
ROC curves of the clinical prediction model for NBNP. **(A)** Training cohort (AUC = 0.816; optimal cutoff = 0.348, sensitivity 96.4%, specificity 54.2%). **(B)** Validation cohort with Bootstrap internal validation (1,000 resamples); Red line: apparent ROC; Gray lines: Bootstrap ROC curves (bias-corrected AUC = 0.846, 95% CI: 0.732–0.959).

*Discrimination.* ROC analysis confirmed favorable discriminative capacity for distinguishing NBNP from BNP (Figure 2). Using the Youden index–derived optimal threshold of 0.348, sensitivity and specificity were 96.4 and 54.2% in the derivation cohort, and 88.2 and 67.9% in the validation cohort, respectively. The AUC reached 0.816 (95% CI: 0.735–0.896) in the derivation cohort (Figure 2A). Because the validation subset was relatively small (*n* = 45), Bootstrap resampling with 1,000 iterations was conducted to obtain a bias-corrected estimate. The resulting AUC was 0.846 (95% CI: 0.732–0.959; Figure 2B), and the overlap between the apparent ROC curve and the Bootstrap-generated ROC distribution confirmed consistent classification ability across resamples.

**Figure 3:**
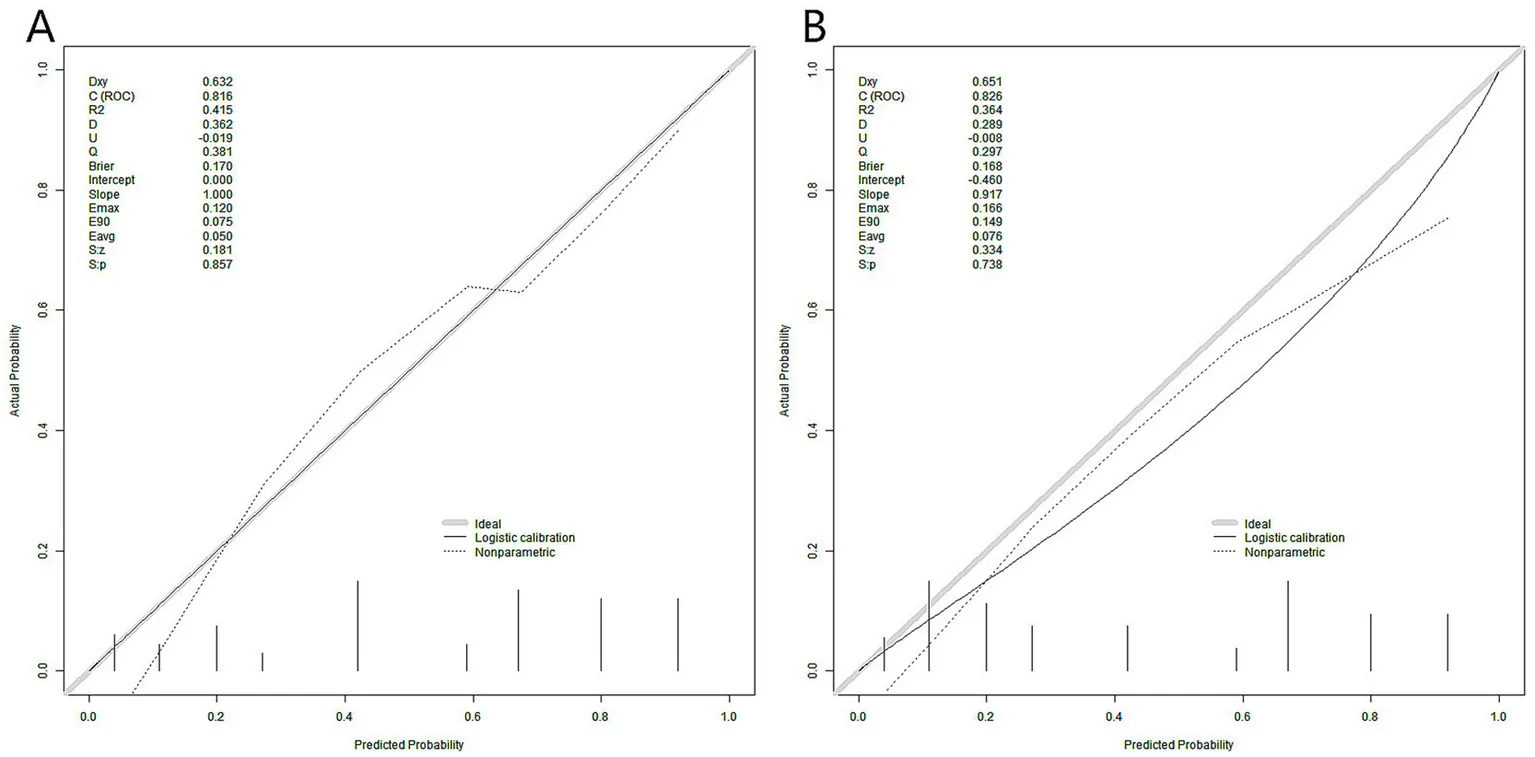
Calibration curves of the clinical prediction model for NBNP **(A)** Training cohort; **(B)** Validation cohort.

*Calibration.* Graphical calibration curves revealed close concordance between model-estimated and empirically observed probabilities in both cohorts (Figure 3), with both logistic and nonparametric fitted curves tracking near the ideal 45° reference line. The Hosmer–Lemeshow test supported adequate fit (derivation cohort *p* = 0.857, Figure 3A; validation cohort *p* = 0.738, Figure 3B), indicating no statistically significant departure of predictions from observed event rates.

### 3.5 Clinical utility

**Figure 4:**
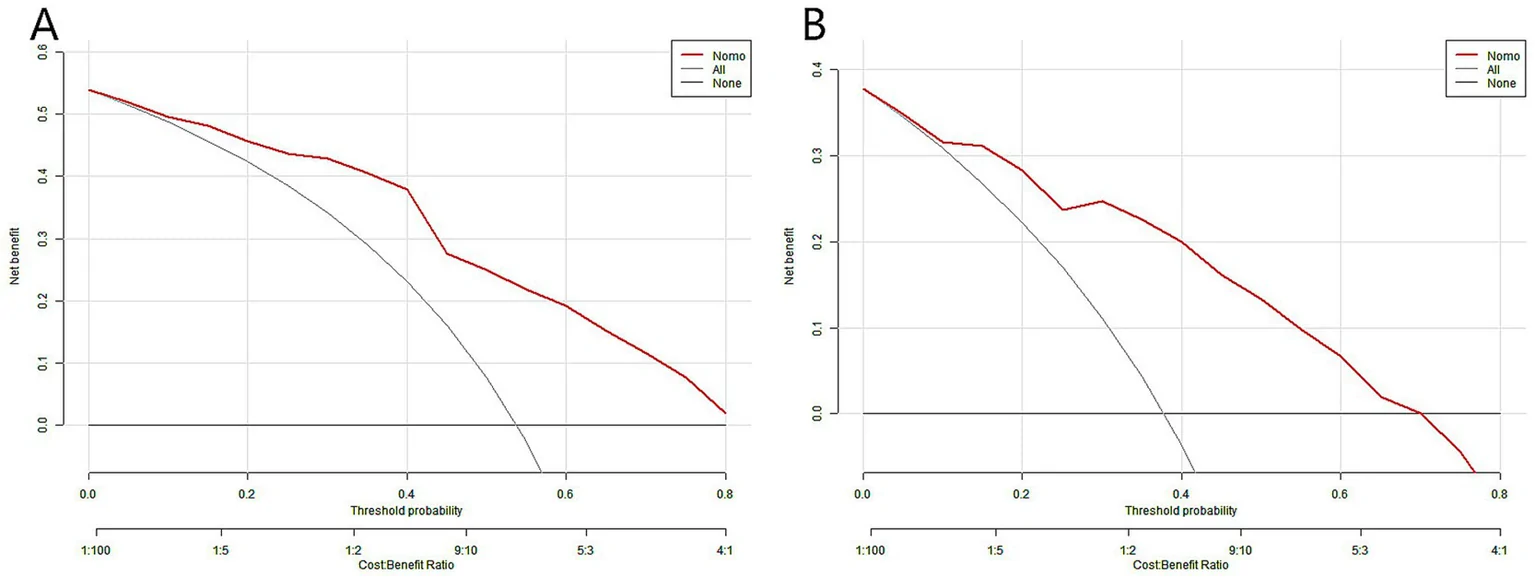
Decision curve analysis (DCA) evaluating the clinical utility of the prediction model for NBNP, demonstrating net benefit across a range of threshold probabilities. **(A)** Training cohort; **(B)** Validation cohort.

DCA was employed to gauge the practical value of applying the nomogram in treatment decisions (Figure 4). Across both cohorts, the nomogram curve consistently exceeded the “treat-all” and “treat-none” reference strategies, yielding positive net benefit over threshold probability ranges of roughly 15–78% (derivation, Figure 4A) and 20–72% (validation, Figure 4B). These findings imply that nomogram-guided decision-making would confer greater clinical benefit than either universal empirical treatment for NBNP or complete omission of empirical therapy, thereby reinforcing the model’s applicability for early etiological triage in pediatric NP.

### 3.6 Sensitivity analysis

When the analysis was confined to the 126 patients whose etiological diagnoses relied solely on BALF culture or tNGS (BNP 62, NBNP 64), all three predictors retained statistical significance. The re-derived model yielded an AUC of 0.905 (95% CI: 0.855–0.955; Supplementary Figure 1), surpassing the primary model and confirming that results were not inflated by possible misclassification inherent to less sensitive sputum-based testing.

## 4 Discussion

In this study, we developed and validated a nomogram for predicting non-bacterial necrotizing pneumonia (NBNP) in children based on three readily accessible parameters: fever duration, white blood cell (WBC) count, and C-reactive protein (CRP) level. Prolonged fever duration was positively associated with NBNP, while elevated WBC and CRP levels favored a bacterial etiology. The nomogram achieved an AUC of 0.816 in the training cohort and a Bootstrap bias-corrected AUC of 0.846 in the validation cohort, with satisfactory calibration and positive net benefit on decision curve analysis. To our knowledge, this is the first prediction model specifically targeting etiological differentiation of established NP.

### 4.1 Clinical need and paradigm shift

NP is an increasingly recognized complication of CAP in children, with a prevalence of approximately 3% (1). While historically attributed to toxin-producing bacteria such as *Streptococcus pneumoniae* and *Staphylococcus aureus* (7), Beyond conventional pyogenic bacteria, atypical and viral pathogens—particularly *Mycoplasma pneumoniae* and adenovirus—are increasingly recognized in pediatric necrotizing pneumonia, reflecting both a broadening etiologic spectrum and improved pathogen detection through molecular diagnostic techniques (7, 8). Management is fundamentally pathogen-dependent: bacterial NP requires aggressive antimicrobial therapy, whereas non-bacterial NP necessitates macrolides combined with corticosteroids and immunomodulatory agents (9). However, blood culture positivity rates remain as low as 10–12%, with results requiring 24–72 h or longer (4). Existing prediction models have focused on identifying which children will progress to NP (5), leaving a gap in etiological classification tools for established NP. Our study addresses this gap by focusing on “what is the likely etiology?” rather than “will NP occur?”—a distinction with direct therapeutic implications.

### 4.2 Pathophysiological basis for WBC and CRP as discriminators

Both WBC and CRP were inversely associated with NBNP, consistent with evidence that typical bacterial pneumonia generally produces more pronounced systemic inflammatory responses than pneumonia caused by viral or atypical pathogens. In a prospective pediatric study, Berg et al. found that increasing CRP levels significantly differentiated bacterial pneumonia from viral/atypical pneumonia, while the combination of CRP > 80 mg/L and an elevated WBC count showed a specificity exceeding 85% for bacterial etiology (10). In our cohort, the median WBC was markedly lower in the NBNP group (6.95 × 10^9^/L) than in the BNP group (14.75 × 10^9^/L), consistent with these patterns.

Regarding CRP, a meta-analysis by Flood et al. of 1,230 children confirmed significantly higher CRP levels in bacterial pneumonia (11), and Don et al. demonstrated that CRP above 80 mg/L strongly suggested bacterial etiology (12). In our study, the NBNP group exhibited lower CRP levels (median 6.80 vs. 14.79 mg/L). Although absolute values in both groups were modest compared with uncomplicated bacterial pneumonia—possibly reflecting the complex inflammatory milieu of necrotizing disease and heterogeneous sampling timing—the relative difference remained clinically meaningful. Our findings extend the discriminatory value of these conventional markers from general CAP to the specific context of established NP.

### 4.3 Fever duration as a discriminator

Fever duration was the strongest predictor of NBNP (OR = 4.580), with the NBNP group exhibiting substantially longer median fever duration (14.00 vs. 8.00 days). This is consistent with the clinical behavior of the predominant non-bacterial pathogens. *M. pneumoniae* characteristically causes protracted febrile illness, with previous studies reporting mean fever durations of 13–21 days in *M. pneumoniae*–associated NP (13, 14). The prolonged fever likely reflects immune-mediated inflammation—including Th1/Th2 dysregulation and excessive cytokine production—rather than direct tissue invasion (15). Adenovirus similarly produces extended febrile courses (16). In contrast, bacterial NP typically presents more acutely, often prompting earlier recognition and intervention, which may account for shorter pre-diagnosis fever duration. Clinically, fever persisting beyond 10 days in a child with confirmed or suspected NP should raise suspicion for a non-bacterial etiology.

### 4.4 Model validation and clinical utility

The discriminative performance of our nomogram (area under the curve (AUC) = 0.816 in training; bias-corrected AUC = 0.846, 95% CI: 0.732–0.959 in validation) compares favorably with related models: nomograms for predicting NP occurrence in refractory *Mycoplasma pneumoniae* pneumonia (RMPP) report AUCs of 0.78–0.85 (5), and models distinguishing bacterial from viral CAP typically achieve AUCs of 0.75–0.85 (17). The Hosmer–Lemeshow test confirmed adequate calibration (*p* = 0.857 and 0.738), and DCA demonstrated net benefit across clinically meaningful threshold ranges. A key practical advantage is that all three constituent variables—fever duration, WBC, and CRP—are universally obtained during initial evaluation, enabling immediate deployment in both tertiary centers and resource-limited settings without additional cost.

### 4.5 Limitations

Several limitations warrant acknowledgment. First, as a single-center retrospective study with a limited sample size (*n* = 149; validation *n* = 45), the risk of overfitting remains despite Bootstrap validation. The relatively wide confidence interval (0.732–0.959) reflects inherent statistical instability that cannot substitute for true external validation. Second, the pathogen spectrum and demographics from a single institution in northern China may limit generalizability. A multicenter prospective validation study is being planned. Third, the etiological reference standard is subject to sensitivity limitations; some NBNP cases may harbor undetected bacterial co-infections. However, a sensitivity analysis restricted to BALF culture– or tNGS-confirmed cases (*n* = 126) yielded an improved AUC of 0.905 (95% CI: 0.855–0.955), confirming robustness against potential misclassification. Finally, the model relies on static admission parameters and does not incorporate dynamic clinical evolution.

## 5 Conclusion

In summary, the nomogram incorporating fever duration, WBC count, and CRP level demonstrated robust discriminative and calibration performance for early etiological differentiation of pediatric NP, offering a practical bedside tool to guide pathogen-directed therapy prior to microbiological confirmation. Given the single-center design and limited sample size, external validation in multicenter prospective cohorts is warranted before clinical implementation.

## Data Availability

The original contributions presented in the study are included in the article/Supplementary material, further inquiries can be directed to the corresponding authors.

## Supplementary material

SUPPLEMENTARY FIGURE 1. ROC curve of the sensitivity analysis restricted to patients with BALF culture- or tNGS-confirmed etiological diagnoses (*n* = 126; AUC = 0.905, 95% CI: 0.855–0.955).

## Glossary

NP: necrotizing pneumonia
CAP: community-acquired pneumonia
BNP: bacterial necrotizing pneumonia
NBNP: non-bacterial necrotizing pneumonia
CT: computed tomography
PCR: polymerase chain reaction
tNGS: targeted next-generation sequencing
BALF: bronchoalveolar lavage fluid
WBC: white blood cell count
CRP: C-reactive protein
N%: neutrophil percentage
Hb: hemoglobin
PLT: platelet count
PCT: procalcitonin
ESR: erythrocyte sedimentation rate
FER: serum ferritin
IgE: Immunoglobulin E
IL-6: interleukin-6
CK: creatine kinase
CK-MB: creatine kinase-MB
ALT: alanine aminotransferase
AST: aspartate aminotransferase
LDH: lactate dehydrogenase
OR: odds ratio
CI: confidence interval
ROC: receiver operating characteristic
AUC: area under the roc curve
DCA: decision curve analysis
EPV: events per variable
RMPP: refractory mycoplasma pneumoniae pneumonia
SD: standard deviation
IQR: interquartile range
